# Fluid–Structure Coupling Effects in a Dual U-Tube Coriolis Mass Flow Meter

**DOI:** 10.3390/s21030982

**Published:** 2021-02-02

**Authors:** Yuh-Chung Hu, Zen-Yu Chen, Pei-Zen Chang

**Affiliations:** 1 Department of Mechanical and Electromechanical Engineering, National ILan University, Yilan 26047, Taiwan; 2 Institute of Applied Mechanics, National Taiwan University, Taipei 10617, Taiwan; r07543004@ntu.edu.tw (Z.-Y.C.); changpz@ntu.edu.tw (P.-Z.C.)

**Keywords:** Coriolis mass flow meter, finite-element simulation, fluid–structure interaction, computational fluid dynamics

## Abstract

Coriolis mass flowmeters are highly customized products involving high-degree fluid-structure coupling dynamics and high-precision manufacture. The typical delay from from order to shipment is at least 4 months. This paper presents some important design considerations through simulation and experiments, so as to provide manufacturers with a more time-efficient product design and manufacture process. This paper aims at simulating the fluid-structure coupling dynamics of a dual U-tube Coriolis mass flowmeter through the COMSOL simulation package. The simulation results are experimentally validated using a dual U-tube CMF manufactured by Yokogawa Co., Ltd. in a TAF certified flow testing factory provided by FineTek Co., Ltd. Some important design considerations are drawn from simulation and experiment. The zero drift will occur when the dual U-tube structure is unbalanced and therefore the dynamic balance is very important in the manufacturing of dual U-tube CMF. The fluid viscosity can be determined from the driving current of the voice coil actuator or the pressure loss between the inlet and outlet of CMF. Finally, the authors develop a simulation application based on COMSOL’s development platform. Users can quickly evaluate their design through by using this application. The present application can significantly shorten product design and manufacturing time.

## 1. Introduction

A Coriolis mass flow meter (CMF) facilitates the direct measurement of mass flow rates. A CMF’s measurement accuracy is high because it is less affected by factors such as the medium’s density, pressure and temperature. In addition to a large flow range and good turndown ratio and repeatability as well as the absence of blocking elements and movable parts in the flow tube, a CMF has a long service life. Moreover, CMFs of varied structural designs can be used for different fluid media, such as high-viscosity and non-Newtonian fluids as well as liquids containing trace gases, under certain conditions. Therefore, CMFs are widely used in various industries, such as chemical, marine, petrochemical, energy, pulp and paper, and water treatment industries. In addition, CMFs can be used to measure volume flow rate, density, temperature, viscosity, and concentration. The industrial demand for flow measurement technology has led to an increase in CMF research and development. Currently, the most popular CMF for industrial applications is the dual U-tube CMF. Thus, the current article focuses on further research and development of the dual U-tube CMF. Because the principle underlying CMF operation involves a complex fluid–structure interaction of structural vibration, this study uses finite element simulation to consider the fluid characteristics, boundary conditions, and complete geometric structure of CMF operation to perform CMF structural analysis and measure fluid density, mass flow rate, and fluid viscosity.

According to the shape of the oscillatory tube, CMFs are commonly classified into the three types: U-tube, Ω-tube, and straight-tube. Wang et al. [1] summarized the technical specifications of commercial CMFs: tube diameter, 1 to 205 mm; nominal flow range, 100 g/h to 2000 t/h; accuracy, 0.05% to 0.5% for liquids and 0.35% to 0.75% for gases; zero stability, 0.002% to 0.1% of nominal flowrate; repeatability is typically half the accuracy; common tube materials include 300-series stainless steel, super duplex, alloy C-22, titanium, tantalum, zirconium, and perfluoroalkoxy alkanes (PFA); operation temperature range, −240 to 427 °C; pressure rating up to 413 bar. When the CMF was first developed around 1950 and further improved later, the fluid–structure coupling problem of the CMF remained extremely complex and only experimental proof of its function could be obtained at that time. However, in 1989, Sultan and Hemp [2] proposed the first detailed mathematical model of a typical U-tube CMF. They modeled a U-tube CMF by using the theory of beam vibration. The simulation result was obtained using the numerical method and validated through experimental measurements performed on a commercial U-tube CMF from Micro Motion. Subsequently, a straight-tube CMF was analyzed by Raszillier and Durst [3]. They considered the tube as a beam and the fluid as a moving string to obtain a simple one-degree-of-freedom system with all the essential features of the CMF. By using the theory of beam vibration and experimental proof, Sultan [4] verified that when the position of the motion sensor of the straight-tube CMF is closer to the fixed end, the greater is the phase difference between the two output signals of the sensors. Stack et al. [5] introduced a finite-element method to solve the equation of motion of the Timoshenko beam for fluid transportation. They also proved the feasibility and importance of numerical methods for modeling CMFs. Kalotay [6] proposed a method for measuring fluid viscosity by using CMFs, where fluid pressure was measured at the CMF inlet and outlet to obtain pipe flow pressure drop. The Hagen–Poiseuille equation was then applied to calculate fluid viscosity; however, this method could only be applied when the flow field was laminar. Keita [7] indicated that fluid–structure interaction is the operating principle of CMF. Although simple coupling effects can be modeled using mathematical models, the mathematical calculations related to computational fluid dynamics and real coupling problems are extremely complex. Therefore, to model practical operating behaviors, Keita used the simulation software program ADINA to perform finite-element analysis for deriving a straight-tube CMF structure that provides efficient results. Belhadj et al. [8] established a numerical model of a CMF by using the finite-element method in ANSYS. Three matrices of mass, stiffness, and damping were described in the model. The results of the aforementioned authors’ finite-element analysis were consistent with their experimental results, which indicated that the finite-element method can provide an accurate numerical simulation for a CMF. Drahm and Bjonnes [9] proposed a method that can use drive current magnitude to estimate fluid viscosity. Kumar et al. [10] analyzed the flow field state for a low Reynolds number (*Re*) and proposed that the flow state at a low *Re* may cause deviation in measurement. Their finite-element simulation indicated that the fluid–structure interaction is caused by the secondary oscillation resulting from the force–shear force interaction. Romanov and Beskachko [11] studied the damping vibration and mechanical energy loss characteristics of CMFs by performing fluid–solid coupling analysis and experiments. In fluid–structure coupling analysis, they used ANSYS to develop a 3D model for finite-element analysis. The experimental and simulation results of the aforementioned authors indicated that the structure and fluid cause system energy loss and that the energy loss caused by the fluid increases when drive amplitude and flow rate increase. An important issue is that the mixed fluids of gas and liquid, the so-called two-phase flow, will cause the measurement errors of a CMF. Recently, Gagliano et al. [12] have proposed a methodology for velocity measurement of tow-phase flow in microchannels based on a low-cost optical signals monitoring setup. This provided the feasibility for CMF to overcome the problem of measuring the two-phase flow.

Coriolis mass flow meters (CMFs) are thus highly customized products whose design and manufacture involves a high-degree fluid-structure coupling dynamics and high-precision manufacturing technology, and it often relies on some rules of thumb and can take at least 4 months from order to shipment. This paper aims to systematically investigate the fluid-structure coupling dynamics of a dual U-tube CMF through COMSOL simulation package and experiment and draws some important design considerations, so as to provide manufacturers with a more time-efficient product design and manufacture process. The simulation results are experimentally validated using a dual U-tube CMF manufactured by Yokogawa Taiwan Co., Ltd. (Taipei, Taiwan) in a TAF certified flow testing factory provided by FineTek Co., Ltd. (New Taipei, Taiwan).

## 2. Experiment Methodology

This section describes the experimental setup. The experiment involves the following five tasks: the geometry, motion sensor and driver materials, and parameterization of the sample CMF; the experiment for fluid density measurement; the experiment for mass flow rate measurement; the experiment for the influence of gravity; and the experiment for structural imbalance.

### 2.1. The Geometry, Motion Sensor and Driver Materials, and Parameterization of the Sample CMF

**Figure 1:**
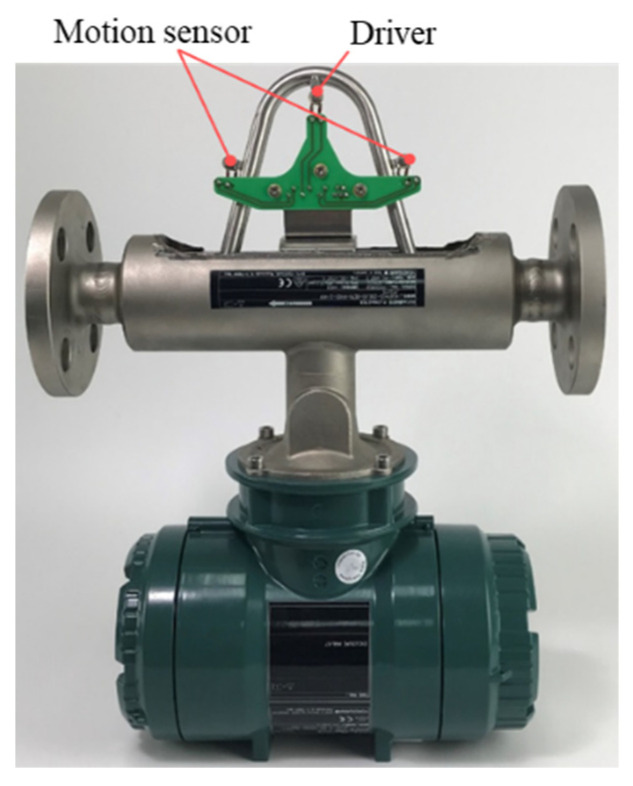
Sample CMF manufactured by Yokogawa Co., Ltd.

**Figure 2:**
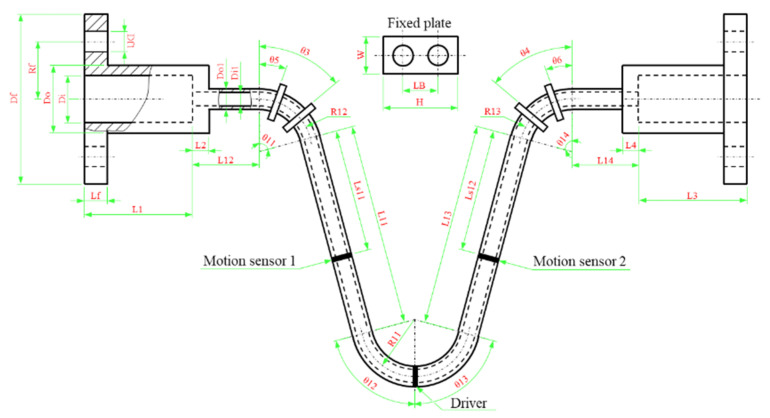
Geometric structure of the CMF.

We measured the geometric dimensions of a sample CMF flow tube (Figure 1), which is manufactured by Yokogawa Co., Ltd. with the maximum measurable mass flow rate 2 kg/s and accuracy 0.2% and the accuracy of density 4 kg/m^3^, and parameterized the flow tube (Figure 2). The dimensions of the flow tube are listed in Table 1, and the motion sensor and driver materials are presented in Table 2.

### 2.2. Experiment for Fluid Density Measurement

**Figure 3:**
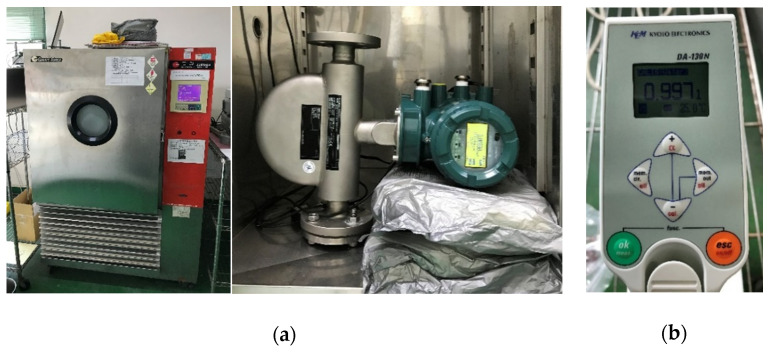
Density experiment setup: (**a**) the demonstration mass flow meter in a constant temperature chamber; (**b**) the densitometer manufactured by Kyoto Electronics Manufacturing Co., Ltd.

The density measurement experiment is conducted under static fluid conditions, that is the fluid does not flow. We prepared various solutions with different weight percentage concentrations (wt%) of sodium chloride to vary the density of the liquid. For the experiments, we sealed one side of the CMF and poured the liquid into it. During the experiment, the CMF was kept in a temperature chamber to maintain the liquid’s temperature at 25 °C ± 0.05 °C, as shown in Figure 3a. If there are bubbles in the flow tube, the fluid in the flow tube will flow slightly, causing a slight phase difference between the two motion sensors (Figure 2). In order to avoid the interference of bubbles in the fluid to the experiment, we must ensure that the phase difference between the two motion sensors has to be less than 0.01 mrad, otherwise, the experiment must be repeated. According to the manufacturing specifications, the density measurement accuracy of CMF is ±4 $\mathrm{kg}/m^{3}$. We used a densitometer, shown in Figure 3b, to measure the fluid density to obtain the standard density for verifying whether the experimental results were satisfactory. The densitometer is manufactured by Kyoto Electronic Manufacturing Co., Ltd. (KEM, Tokyo, Japan) with measurement accuracy ±1 kg/m^3^ and resolution 0.1 kg/m^3^. The densities measured by the sample CMF and the driving frequencies and phase difference are captured through RS485 and Modbus.

### 2.3. Experiment for Mass Flow Rate Measurement

**Figure 4:**
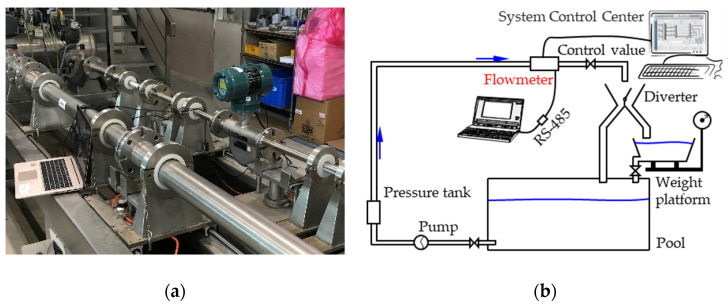
Mass flow rate experiment: (**a**) the experiment setup; (**b**) the schematic of experiment setup.

The mass flow rate experiment was performed in a TAF certificated flow testing factory (FineTek) with the maximum flow speed 50 m^3^/h and accuracy 2%. The CMF was set up in a pipeline, as shown in Figure 4a. The complete experimental facility and schematic are shown in Figure 4b. For precise calculation, the experiment was performed five times for each flow measurement. The measurement results of the CMF were then compared with those of an electronic scale to confirm the accuracy of the experiment.

### 2.4. Experiment for the Influence of Gravity

**Figure 5:**
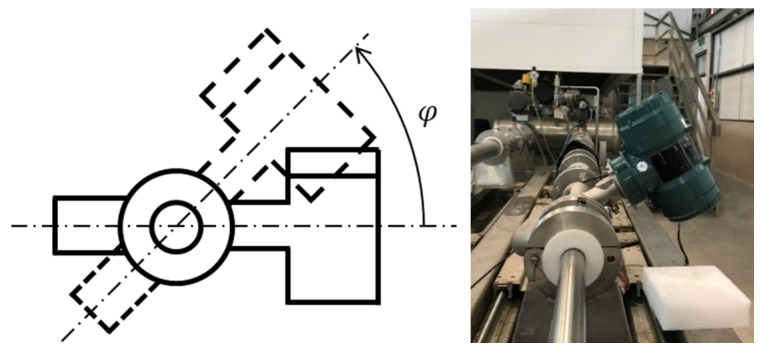
Schematic and photo of the installation of a dual U-tube CMF with a deflection angle.2. Simulation and Experiment.

In a factory, production lines typically include many complicated pipelines. Therefore, the installation angle of CMF must be adjusted according to the actual allowable space. However, the flow tube should be installed vertically downward as far as possible. A guideline is that the flow tube cannot be placed upwards, because air is lighter then water, otherwise the air will accumulate in the flow tube and cause measurement error. To verify whether gravity affects the operation of a dual U-tube CMF and whether installing the CMF with a deflection angle due to the aforementioned reasons is essential, Figure 5 illustrates the schematic of the experiment, in which the angles of deflection (φ) were 30°, 45°, 60°, 90°, 120°, 135°, and 150°.

### 2.5. Experiment for Structural Imbalance

**Figure 6:**
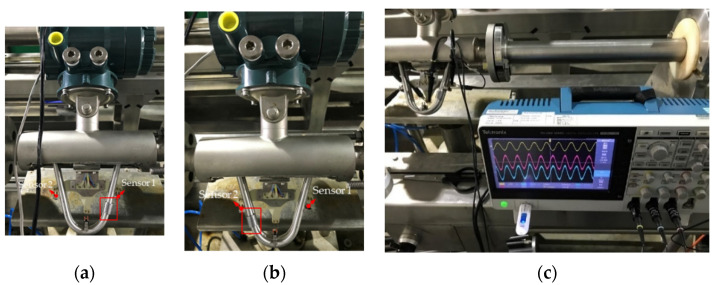
Setup of the structural imbalance experiment: (**a**) mass added 10 mm below only Sensor 1, (**b**) mass added 10 mm below only Sensor 2, and (**c**) an oscilloscope used to capture sensor output signals.

A CMF can become structurally imbalanced due to manufacturing tolerances, material unevenness, poor weight design, and other issues. Therefore, in the experiment, we added mass to the flow tube, introduced structural imbalance, and observed the motion sensor output signals. The simulation results were compared with the experimental results to confirm the consistency between them. The obtained results were used to verify the utility of COMSOL-based finite-element simulation to observe structural imbalance during the product development process. The experiment setup is displayed in Figure 6. We paste the lead strip around the flow tube to serve as an additional mass. To prevent the additional mass from hitting another flow tube when oscillation, the allowable thickest lead strip is 10-g. However, it is almost two times the motion sensor’s mass and also heavier than the driver’s mass. The additional mass was added 10 mm below Sensors 1 and 2, respectively, and the output voltage signals of these motion sensors were captured using an oscilloscope. In the experiment, the flow rate was fixed at approximately 1 kg/s.

## 3. Simulation Methodology

This section describes the methodology and the theoretical basis involved in the simulation of the structure-fluid coupling dynamics of a dual U-tube CMF through finite-element modeling on COMSOL, followed by experimental verification of the simulation results. The simulation contains the following four tasks, which will be included in the application (App) developed by the authors to simulate the performance of a dual U-tube CMF: simulation on the fluid density measurement of a CMF; simulation on the flow rate measurement of a CMF; simulation on the influence of gravity on the performance of a CMF; and simulation on the influence of structural imbalance on the performance of a CMF.

**Figure 7:**
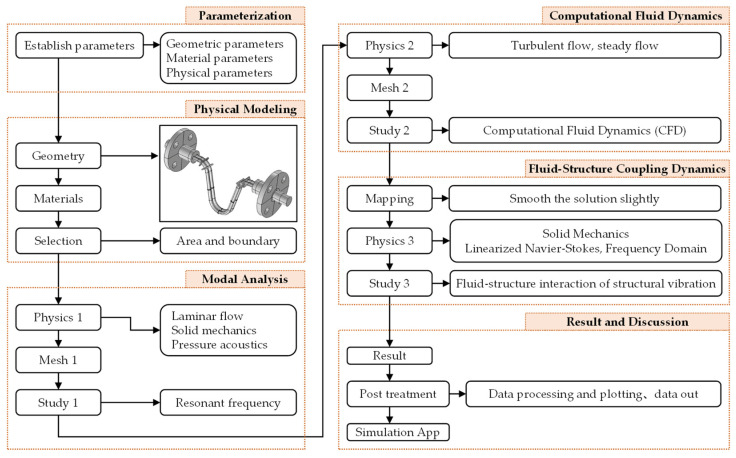
Simulation steps for a CMF.

The simulation flowchart (Figure 7) can be divided into the following 6 major steps: parameterization, physical modeling, modal analysis, computational fluid dynamics (CFD), fluid-structure coupling dynamics, and result and discussion. Eventually, the whole simulation tasks will be written as a simulation App based on the COMSOL development platform. The parameterization process has been done in the experiment, refer to the Section 2.1 and results are shown in Figure 2, Table 1 and Table 2. The remaining 5 major steps will be introduced in the following subsections.

### 3.1. Physical Modeling

**Figure 8:**
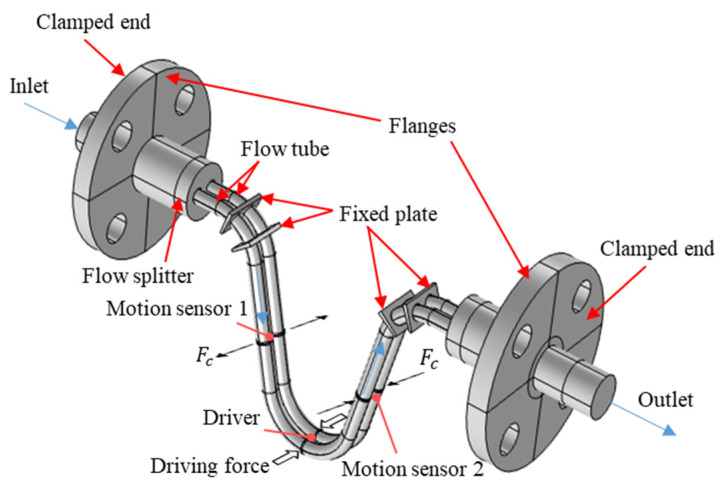
The physical model of the CMF.

This major step can further be divided into three substeps: geometry, materials, and selection. The first two substeps use COMSOL’s built-in drawing program to build the physical model of CMF based on the characteristic parameters of the previous major step. (Figure 8). The selection step is to define the areas of solid and fluid and set the boundary conditions at flow tubes’ inlet and outlet and wall. The motion sensors and driver are assumed to be concentrated mass. The surfaces of the flanges are assumed to be fixed boundaries. The driver exerts a harmonic driving force on the middle point of the flow tube.

### 3.2. Modal Analysis

This major step is to analyze the relationship between the resonant frequencies and fluid densities of the flow tube containing static fluid and can be further divided into 3 sub-steps: physics 1, mesh 1, and study 1. Physics 1 involves laminar flow, solid mechanics, and pressure acoustics. Since the fluid is static, then, to save numerical calculation resources, it does not need to mesh the boundary layer of the fluid at Mesh 1. Study 1 is to determine the resonant frequencies of the flow tube containing static fluid and the resulting fundamental frequency will be the driving frequency at the fluid-structure dynamics.

### 3.3. Computational Fluid Dynamics

Before the fluid-structure coupling dynamics, the flow field inside the flow tube has to be solved. This major step is to analyze the distributions of velocity, pressure, and shear stress of the flow field inside the flow tube and can be further divided into 3 sub-steps: physics 2, mesh 2, and study 2. Physics 2 involves computational fluid dynamics (CFD). The Reynolds-Averaged Navier–Stokes Equations (RANS) was used to determine the turbulent flow field in the flow tube based on the following four assumptions:

- The fluid is assumed to be a Newtonian fluid because the fluid in the experiment is water;
- The fluid is assumed to have an incompressible flow because the ratio of flow velocity to sound velocity for the fluid is a Mach number less than 0.3;
- Assume steady-state flow field, namely the velocity field is time-invariant, because all the measurements in the flow experiments are performed after the steady-state is reached;
- The fluid density, *ρ_f_*, is constant because the experiment is kept in a constant temperature chamber to maintain the liquid’s temperature at 25 °C ± 0.05 °C;
- The influence of gravity can be ignored during simulation.

RANS considers the flow field to be the superposition of a time-average term and a fluctuation term, namely the velocities *u_j_* in Cartesian coordinates and the pressure, *p*, can be expressed as:

(1) $$u_{j}={\bar{u}}_{j}+u_{j}^{\prime }\;\mathrm{and}\;p=\bar{p}+p^{\prime }$$

where the over-bar means the time average and the prime means the instant pulsation. Based on the above assumptions, the continuity equation, momentum equation, and energy equation are expressed in Einstein notation and in Cartesian coordinates as, respectively:

(2) $$\frac{\partial u_{j}}{\partial x_{j}}=0$$

(3) $$u_{j}\frac{\partial u_{i}}{\partial x_{j}}=-\frac{1}{\rho_{f}}\frac{\partial p\delta_{ij}}{\partial x_{j}}+\nu \frac{\partial^{2}u_{i}}{\partial x_{j}^{2}}-\frac{\partial \bar{u_{i}^{\prime }u_{j}^{\prime }}}{\partial x_{j}}+F_{i}$$

(4) $$\rho_{f}C_{p}u_{j}\frac{\partial T}{\partial x_{j}}=\beta Tu_{j}\frac{\partial p}{\partial x_{j}}+K\frac{\partial^{2}T}{\partial x_{j}^{2}}+X$$

where ν is dynamic viscosity, *C_p_* is the specific heat at constant pressure, *β* is the thermal expansion coefficient of fluid, *T* is temperature, *K* is the thermal conductivity of fluid, and *X* is the dissipation function given by [13].

(5) $$\begin{array}{c} X=\mu [2{\left(\frac{\partial u_{1}}{\partial x_{1}}\right)}^{2}+2{\left(\frac{\partial u_{2}}{\partial x_{2}}\right)}^{2}+2{\left(\frac{\partial u_{3}}{\partial x_{3}}\right)}^{2}+{\left(\frac{\partial u_{2}}{\partial x_{1}}+\frac{\partial u_{1}}{\partial x_{2}}\right)}^{2}\\ +{\left(\frac{\partial u_{3}}{\partial x_{2}}+\frac{\partial u_{2}}{\partial x_{3}}\right)}^{2}+{\left(\frac{\partial u_{1}}{\partial x_{3}}+\frac{\partial u_{3}}{\partial x_{1}}\right)}^{2}-\frac{2}{3}{\left(\frac{\partial u_{1}}{\partial x_{1}}+\frac{\partial u_{2}}{\partial x_{2}}+\frac{\partial u_{3}}{\partial x_{3}}\right)}^{2}] \end{array}.$$

**Figure 9:**
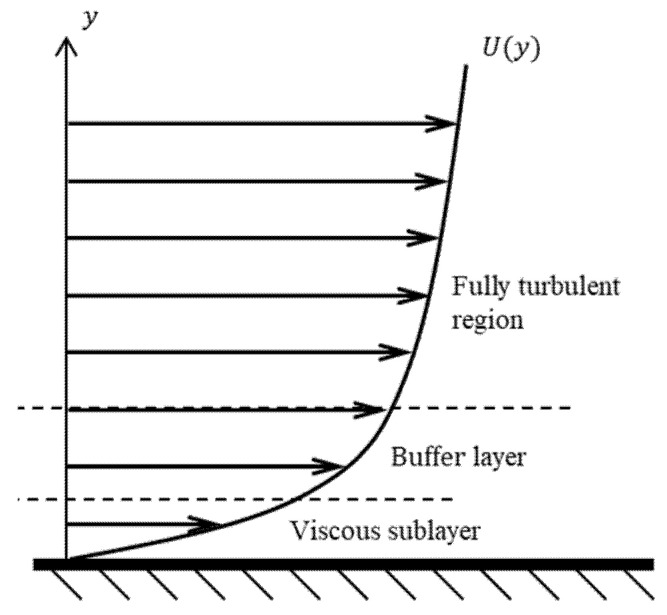
The schematic of turbulent flow field.

The flow field inside the flow tube is usually turbulent flow. The turbulent flow field can be roughly divided into three layers: viscous sublayer, buffer layer, and fully turbulent region (Figure 9). Therefore, the mesh 2 step has to consider the boundary layer. To solve the turbulence field, this study used two turbulence models commonly used in the industry: the *k-ε* and shear stress transport (SST) turbulence models.

The *k-ε* turbulence model, a quasi-empirical model, was proposed by Jones and Launder [14], where *k* means the turbulence kinetic energy and *ε* means the turbulence dissipation rate. It does not solve in detail the flow field with a large velocity gradient in the boundary layer but uses a wall-function to approximate a non-zero flow field on the wall. The *k-ε* turbulence model has a better convergence rate and less computing resources, but it cannot accurately solve more complex flow fields, such as reverse pressure gradients, jets, and strong curvature flow fields. The k*-ε* turbulence model requires fewer computational resources and a shorter computational time than does the SST turbulence model. Nevertheless, the k*-ε* turbulence model ignores the flow field of the buffer layer and viscous sublayer and analytically computes a nonzero fluid velocity at the wall with a wall function. The k*-ε* turbulence model is derived based on the assumption that the flow field is only fully turbulent region, in which the turbulent kinetic energy, *k*, and the dissipation rate of the kinetic energy, *ε*, should satisfy the following two equations, namely the turbulent kinetic energy equation and the kinetic energy dissipation rate equation, respectively:

(6) $$\frac{\partial (\rho_{f}k)}{\partial t}+\frac{\partial (\rho_{f}ku_{i})}{\partial x_{i}}=\frac{\partial }{\partial x_{j}}\left[\left(\mu +\frac{\mu_{t}}{\sigma_{k}}\right)\frac{\partial k}{\partial x_{j}}\right]+P_{k}-\rho_{f}\varepsilon$$

(7) $$\frac{\partial (\rho_{f}\varepsilon )}{\partial t}+\frac{\partial (\rho_{f}\varepsilon u_{i})}{\partial x_{i}}=\frac{\partial }{\partial x_{j}}\left[\left(\mu +\frac{\mu_{t}}{\sigma_{\varepsilon }}\right)\frac{\partial \varepsilon }{\partial x_{j}}\right]+\frac{\varepsilon }{k}(C_{1\varepsilon }P_{k}-C_{2\varepsilon }\rho_{f}\varepsilon )$$

where *μ_t_*, turbulent viscosity, and *P_k_*, the production term that is the kinetic energy generated by buoyancy and laminar velocity gradient, are given by

(8) $$\mu_{t}=\rho_{f}C_{\mu }\frac{k^{2}}{\varepsilon },\;P_{k}=\rho_{f}\tau_{ij}\frac{\partial u_{i}}{\partial x_{j}}$$

where *τ_ij_* is Reynolds shear tensor and the constants in Equations (6)–(8) is listed in Table 3.

The SST turbulence model, proposed by Menter [15], uses a low *Re* model to solve the accurate boundary layer flow field; however, it requires sufficient mesh refinement. According to the law of the wall, the thickness of the first layer of mesh, *Y*, is expressed as follows:

(9) $$Y=\frac{Y^{+}\mu }{\rho_{f}U_{\tau }}$$

where $Y^{+}$ is the dimensionless wall distance, $U_{\tau }$ is the friction velocity, $\rho_{f}$ is the fluid density, and μ is the fluid viscosity.

(10) Uτ=τw/ρf, τw=ρfCfU2/2, Cf=0.079Re−0.25

where *C_f_* is Fanning friction factor. According to previous experimental studies, if the boundary layer flow field is to be determined accurately, the dimensionless wall distance should be approximately 1. The ranges of the viscous sublayer and buffer layer are approximately $0\leq Y^{+}\leq 5$ and $5\leq Y^{+}\leq 30$, respectively. If set the mesh growing rate to 1.2, then Y+ will be greater than 30 when the boundary layer mesh to 11 layers. SST turbulence model calculates the flow field of the boundary through combining the *k-ε* and *k-ω* models, where *k-ε* model is a free-flow model while *k-ω* model is a near-wall model. The ω of *k-ω* model means specific dissipation rate. The transport equation of *k* and *ω* are given by, respectively:

(11) $$\frac{\partial (\rho k)}{\partial t}+\frac{\partial (\rho ku_{j})}{\partial x_{j}}=P_{k}-\rho \beta_{0}^{*}\omega k+\frac{\partial }{\partial x_{j}}\left[(\mu +\sigma_{k1}\mu_{t})\frac{\partial k}{\partial x_{j}}\right]$$

(12) $$\frac{\partial (\rho \omega )}{\partial t}+\frac{\partial (\rho \omega u_{j})}{\partial x_{j}}=\frac{\rho \alpha_{1}}{\mu_{t}}P_{k}-\rho \beta_{1}\omega^{2}+\frac{\partial }{\partial x_{j}}\left[(\mu +\sigma_{\omega 1}\mu_{t})\frac{\partial \omega }{\partial x_{j}}\right]$$

Herein, the *k-ε* model has to rewritten as transformed *k-ε* model:

(13) $$\frac{\partial (\rho k)}{\partial t}+\frac{\partial (\rho ku_{j})}{\partial x_{j}}=P_{k}-\rho \beta_{0}^{*}\omega k+\frac{\partial }{\partial x_{j}}\left[(\mu +\sigma_{k2}\mu_{t})\frac{\partial k}{\partial x_{j}}\right]$$

(14) $$\frac{\partial (\rho \omega )}{\partial t}+\frac{\partial (\rho \omega u_{j})}{\partial x_{j}}=\frac{\rho \alpha_{2}}{\mu_{t}}P_{k}-\rho \beta_{2}\omega^{2}+\frac{\partial }{\partial x_{j}}\left[(\mu +\sigma_{\omega 2}\mu_{t})\frac{\partial \omega }{\partial x_{j}}\right]+2\frac{\rho \sigma_{\omega 2}}{\omega }\frac{\partial k}{\partial x_{j}}\frac{\partial \omega }{\partial x_{j}}$$

Symbolizing the *k-ε* and *k-ω* models as Ψ_1_ and Ψ_2_ respectively and combining the two models through the tuning function *F*_1_ results in the SST model, Ψ, namely:

(15) $$\Psi =F_{1}\Psi_{1}+(1-F_{1})\Psi_{2}$$

When *F*_1_ is 1, it is the boundary layer flow field, when *F*_1_ is 0, it is the free flow field. Since *k-ε* and *k-ω* models are quasi-empirical models, then some constants are also appear in SST model, as listed in Table 4.

**Figure 10:**
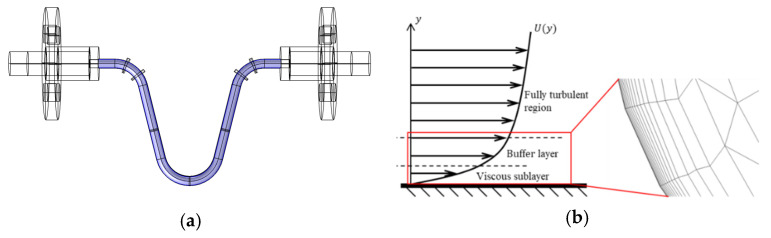
The mesh of flow field: (**a**) the region of boundary layer mesh; (**b**) the boundary layer mesh.

**Figure 11:**
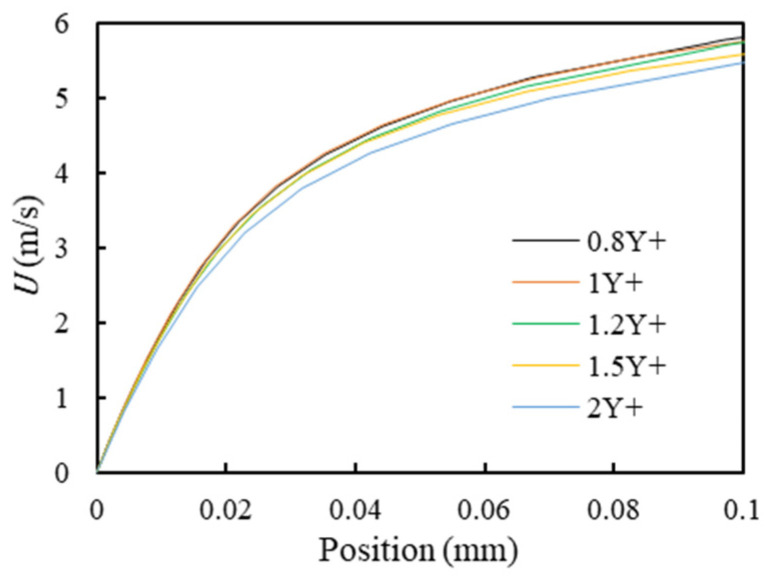
The convergence analysis of the boundary mesh.

Since the CMF oscillates only in the U-tube region, namely the blue-colored region shown in Figure 10a, then the boundary layer mesh is only in that region and the mesh growing rate is set to be 1.2. Figure 10b shows the schematic of boundary layer mesh. As mentioned above, to calculate the boundary layer flow field accurately, the dimensionless wall distance should be approximately 1. Therefore, the convergence analysis of mesh is conducted to determine how many times Y^+^ the first mesh layer has to be. The convergence is judged by the flow velocity at the middle point of the flow tube. Figure 11 shows that the thickness of the boundary layer mesh converges at 1Y^+^ and thereby will be adopted in the following simulation.

### 3.4. Fluid-Structure Coupling Dynamics

This major step can be further divided into three sub-steps: mapping, physics 3, and study 3. The operation principle of CMF involves fluid–structure coupling vibration problems due to the interaction between the fluid and the flow tube. A small amplitude, which is much smaller than the dimensions of the flow tube, and rapid vibration of the flow tube lead to the small and rapid changes in speed, pressure, and density in the flow field. These rapid changes will cause the pressure waves (or sound waves) in the fluid, so energy is transferred in the form of pressure waves (or sound waves) at the fluid–solid interface. Thus, the fluid–structure coupling vibration problems can be considered to be caused by a fluid-structure coupling in the frequency domain. Because COMSOL uses acoustic-structure coupling to solve the problem of fluid-structure coupling vibration, and the acoustic field is solved by Linearized Navier-Stokes Equations (LNS), while, as mentioned in the previous subsection, the flow field is solved by Reynolds-Averaged Navier–Stokes Equations (RANS), therefore the mapping from flow field into acoustic field is required at this step.

Here is a brief introduction to why use the acoustic-structure coupling to solve the fluid-structure coupling vibration problem of CMF. Based on the assumption of small vibration and neglecting fluid viscosity, the continuous equation and linearized Euler equation of motion are respectively expressed as:

(16) $$\frac{\partial \rho_{f}}{\partial t}+\rho_{f}\nabla \cdot \vec{U}=0\;\mathrm{and}\;-\nabla p=\rho_{f}\frac{\partial \vec{U}}{\partial t}$$

The above two equations can be integrated as:

(17) $$\nabla^{2}p=\frac{\partial^{2}\rho_{f}}{\partial t^{2}}$$

Based on the equation of state of ideal gas, the fluid pressure can be expensed with respect to the density by Tayler’s series, namely the fluid compressibility, and neglect the higher-order terms based on the assumption of small vibration:

(18) $$p-p_{0}\approx \frac{\partial p}{\partial \rho_{f}}(\rho_{f}-\rho_{f0})$$

where $p_{0}$ and $\rho_{f0}$ are the fluid pressure and density at the equilibrium state, respectively. Substituting the sound speed, $c_{0}=\partial p/\partial \rho_{f}$, into the fluid compressibility yields

(19) $$p-p_{0}\approx c_{0}{}^{2}(\rho_{f}-\rho_{f0})$$

and it is shown that sound speed means the compressibility of fluid, the higher the sound speed, the stronger the incompressibility of the fluid. The sound speed of water at room temperature is about 1490 m/s, therefore water has a considerable degree of compressibility and must be taken into consideration in simulation. Substituting Equation (18) into Equation (16) yields the acoustic wave equation:

(20) $$\frac{\partial^{2}p}{\partial t^{2}}-c_{0}{}^{2}\nabla^{2}p=0$$

The flow behavior of non-spinning and non-viscous fluid can be described in terms of the velocity potential function, φ, namely $\vec{U}=\nabla \varphi$. Substituting the velocity potential function into Equations (16) and (20) gives the relationship between pressure and velocity potential and the wave equation in terms of velocity potential, respectively:

(21) $$p=-\rho_{f}\frac{\partial \varphi }{\partial t}\;\mathrm{and}\;\frac{\partial^{2}\varphi }{\partial t^{2}}-c_{0}{}^{2}\nabla^{2}\varphi =0$$

Eventually, expressing the velocity potential function in frequency domain, namely $\varphi =i\omega \Phi e^{i\omega t}$, gives the flow velocity, $\vec{V}$, and fluid pressure, *P*, in frequency domain, respectively:

(22) $$\vec{V}=i\omega \nabla \Phi \;\mathrm{and}\;P=\rho_{f}\omega^{2}\Phi$$

## 4. Results and Discussions

This section presents and compares the results of experiments and simulations. The results are obtained based on the methodologies of experiment and simulation described in Section 2 and Section 3, respectively.

### 4.1. Resonant Frequency vs. Fluid Density

**Figure 12:**
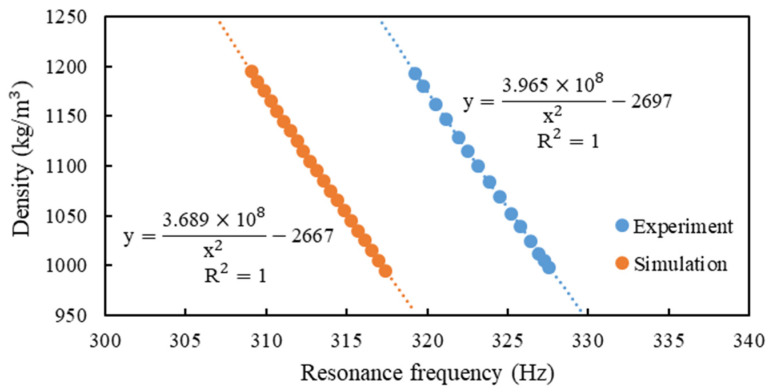
Density–resonance frequency relationship in the simulation and experiment.

Table 5 presents the results of experiment and simulation for the resonant frequencies of CMF with different fluid densities. For the measurement of densities, the absolute errors were within the specification claimed by the manufacturer, namely less than 4 $\mathrm{kg}/m^{3}$. The phase differences, all less than 0.01 mrad, show that the effect of bubbles in the fluid is negligible. Therefore, the experiment is reliable. The results of simulation and experiment are visualized in Figure 12. It can be seen that the fluid density is inversely proportional to the square of resonant frequency. The simulation agrees very well with the experiment though there is a regular deviation of about only 3% between the resonant frequency of experiment and simulation for different fluid density. This regular deviation maybe comes from the frequency-dependent dynamic water stiffness and damping and the mass density of the solid-air-water mixture [16]. Therefore, this finite element modeling can effectively simulate the resonance frequency of CMF when filled with static fluid. R^2^ in the Figure 12 means the coefficient of determination of curve fitting, whose value is between 0 and 1. The closer R^2^ is to 1, the better the regression equation fits; the closer R^2^ is to 0, the worse the regression equation fits. Both the regression equations of experiment and simulation are equal to 1.

### 4.2. Signal Time-Difference vs. Mass Flow Rate

**Figure 13:**
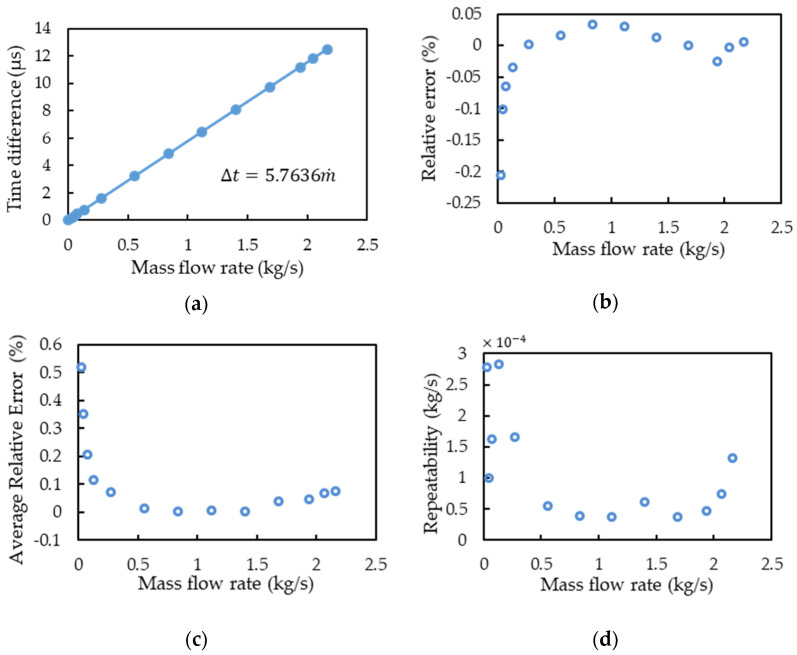
Mass flow rate experiment results: (**a**) the time difference of output signals vs. the mass flow rate; (**b**) the relative error analysis of linear regression; (**c**) the average relative error analysis of linear regression; (**d**) the CMF measurement repeatability analysis.

Table 6 lists the results of simulation and experiment for the signal time-difference of the motion sensors with different mass flow rates and the data are visualized in Figure 13. For precise calculation, the experiment was performed five times for each flow measurement. Figure 13a showed the time differences of output signals with respect to mass flow rate and the linear regression. Figure 13b showed the residual analysis to investigate the linear regression quality, except for the lower flow rate, others can be <0.05%. The relative error was calculated by:

(23) $$E_{r}=\frac{y-y^{\prime }}{y}\times 100\%$$

where *E_r_* is relative error, y is the measured value, and $y^{\prime }$ is the prediction value. As illustrated in Figure 13c, the average relative error of the CMF measurement was within 0.2%, except at a low flow rate. Figure 13d showed that the CMF measurement repeatability was also relatively high. The phase difference–mass flow rate relationship is nonlinear when the flow rate is low possibly because of the zero drift or because small *Re* values lead to flow measurement errors [10].

**Figure 14:**
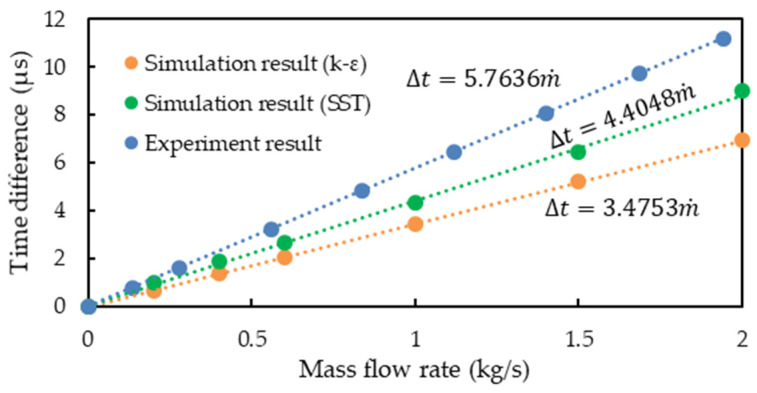
Mass flow rate–phase difference relationship in the simulation and experiment.

The simulation and experimental results of the relationship between the mass flow rate and phase difference are illustrated in Figure 14, where the simulation results of the SST turbulence model are closer to the experimental results than those of the k*-ε* turbulence model. This is due to the *k-ε* turbulence model ignores the flow field of buffer layer and viscous sublayer and analytically calculate a nonzero fluid velocity at the wall by means of a wall function; while the SST turbulence model uses a low Reynolds number model to calculate the accurate boundary layer flow field and the fluid velocity at the wall is zero. The k*-ε* turbulence model requires fewer computational resources and a shorter computational time than does the SST turbulence model. Therefore, in the current case, accurate calculation of the boundary layer is essential to analyze the fluid–structure coupling simulation of the CMF accurately. The relative error between the simulation results of the SST turbulence model and the experimental results was approximately 23.5%. Though the current SST turbulence model is not accurate enough, it reveals that accurate calculation of the boundary layer is essential to analyze the fluid–structure coupling simulation of the CMF accurately, but accurate calculation of the boundary layer requires very fine mesh and very large numerical computation loading.

### 4.3. The Influence of Gravity

**Figure 15:**
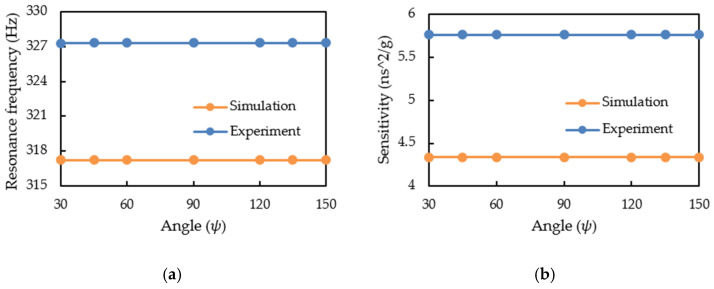
The influence of installation with a deflection angle on: (**a**) CMF resonance frequency; (**b**) CMF sensitivity.

Figure 15 visualizes the results of experiment and simulation regarding the influence of gravity on the performance of CMF. The deflection angle did not affect the resonance frequency and sensitivity of the CMF. Thus, the measured fluid density and mass flow rate were not affected by gravity. When CMF measures fluid, it can install any deflection angle under the premise that the flow tubes are oriented downward.

### 4.4. The Influence of Structural Imbalance

**Figure 16:**
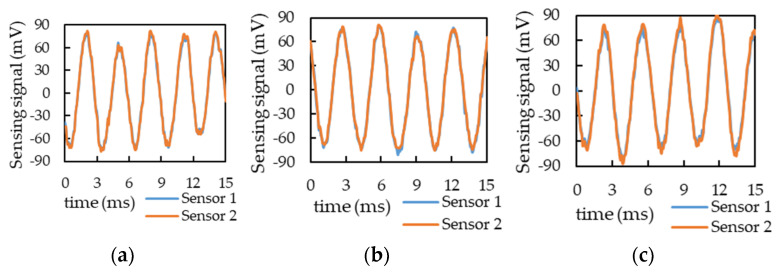
The raw output voltage signals of the two motion sensors in the first 15 ms: (**a**) no additional mass, (**b**) with an additional mass below Sensor 1, and (**c**) with an additional mass below Sensor 2.

Figure 16 displays the raw output voltage signals of the two motion sensors in the first 15 ms: (a) without additional mass; (b) with additional mass below Sensor 1; and (c) with additional mass below Sensor 2, respectively. For both the sensors, the output signals changed; therefore, the raw time-domain signal was changed to a periodic signal and then a fast Fourier transform was applied to observe the changes in the signal frequencies and amplitudes.

Table 7 presents the average amplitude values of five experiments. Ideally, when no additional mass, the voltage signal amplitudes of Sensors 1 and 2 should be identical; however, in the experiment, the voltage signal amplitude of Sensor 2 was higher than that of Sensor 1 by approximately 2.15 mV. This may be because we have disassembled the sensors and repaired the broken coil and then reassembled it, which may cause the structure to be unbalanced. However, a comparison of the two sensors’ output signals when no additional mass was used indicated that the difference in the imbalanced structure caused changes in the sensors’ output signal amplitudes. The signal amplitude of Sensor 1 was larger than that of Sensor 2 when the additional mass was below Sensor 1, whereas the signal amplitude of Sensor 2 was larger than that of Sensor 1 when the additional mass was below Sensor 2. Moreover, after deducting the offset when no mass was added, the signal amplitude difference and amplitude change were approximately 3 mV and 4% when the mass was added, respectively.

**Figure 17:**
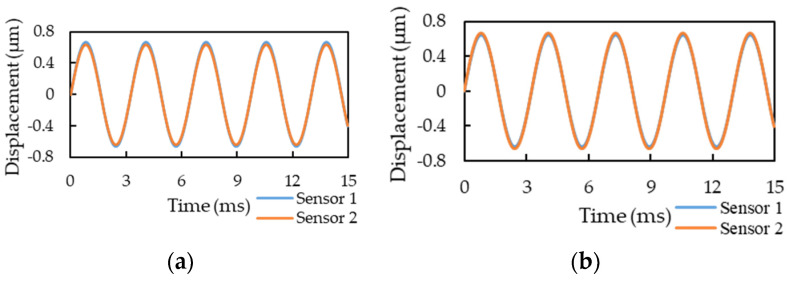
COMSOL-based simulation of displacement signals when a 10-g mass is added below (**a**) Sensor 1 and (**b**) Sensor 2.

The COMSOL simulation added the corresponding mass on the surface of the flow tube according to the additional mass’s magnitude and position in the experiment, and the mass flow rate was 1 kg/s. Figure 17a,b present the signal displacement when the external mass was below Sensor 1 and Sensor 2, respectively. The finite-element simulation results of a sensing displacement signal were consistent with the experimental results. The amplitude of the displacement signal of Sensor 1 was approximately 0.024 μm larger than that of Sensor 2 when the mass was added below Sensor 1, whereas the amplitude of the displacement signal of Sensor 2 was approximately 0.023 μm larger than that of Sensor 1 when the mass was added below Sensor 2. The amplitude change between the two displacement signals was approximately 3.5% compared with the displacement signal without an added mass.

**Figure 18:**
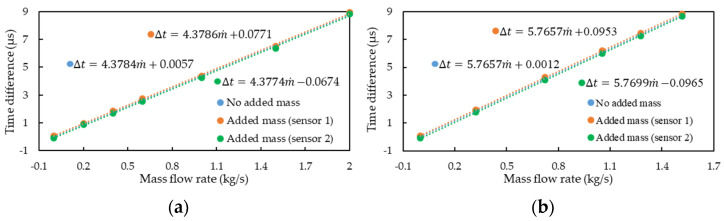
The mass flow–phase difference relationship in an imbalanced CMF structure: (**a**) the simulation results; (**b**) the experimental results.

The imbalanced vibration of the flow-tube structure may cause mass flow measurement error. The principle underlying mass flow measurement is obtained through the phase difference conversion of the two output signals of the motion sensors of the CMF. Through COMSOL finite-element simulation, Figure 18a, and experiment, Figure 18b, we observed that the two output signals exhibited phase differences. The simulation and experimental results indicated that when the three conditions, namely the conditions shown in Figure 6a,b and without additional mass, were fixed at the same flow rate, the addition of an additional mass below Sensors 1 and 2 led to increases and decreases in the phase differences between the two sensor output signals, respectively. However, the addition of mass of different positions only produces an offset in the relationship between the mass flow rate and the signal time difference, does not change the sensitivity, and produces a nonlinear phase difference–mass flow relationship. The reason for these observations was that the additional mass was probably not sufficiently large to affect the dominant frequency and vibration behavior of the flow tube.

## 5. Some Other Design Considerations by Simulation

The simulation tasks mentioned in Section 3 are written as a simulation App, whose user interface is briefed in Appendix A, for dual U-tube CMF based on COMSOL environment. This simulation App provides the users an easier way to evaluate the performance of their design rapidly and therefore drastically shorten the time-span of product development or from order to shipment. In addition to the simulation tasks mentioned in Section 3, some other design considerations can also be drawn from the simulation App, such as fluid viscosity measurement, motion sensor position, and flow splitter design.

### 5.1. Fluid Viscosity Estimation Through Pressure Drop

**Figure 19:**
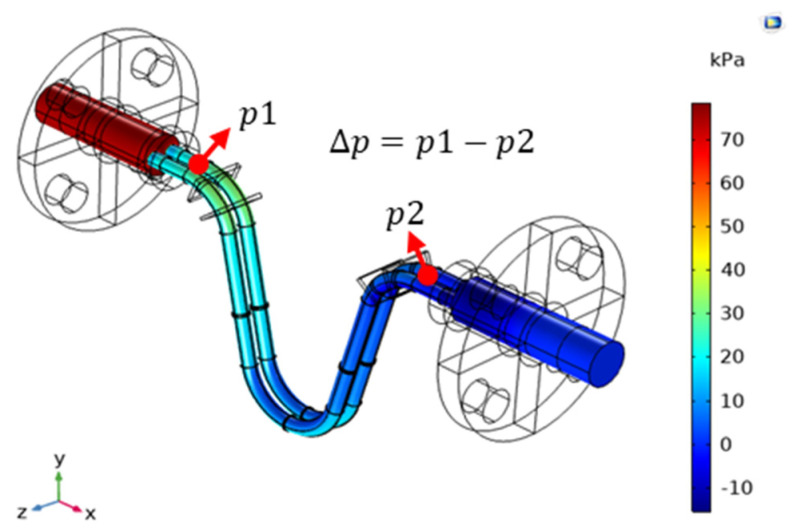
COMSOL-based simulation of the pressure drop of a dual U-tube CMF.

The higher the fluid viscosity, the higher is the shear of the pipe wall, which causes changes in the pipe flow pressure drop. This principle can be used to measure fluid viscosity. In our analysis, we simulated the given fluid’s viscosity by using COMSOL and then observed the pipe flow pressure drop. The pressure drop distribution is displayed in Figure 19. The pressure range between the two ends of the U pipe was measured. Finally, curve fitting was used to determine the relationship between fluid viscosity and the pipe flow pressure drop, and the curve fitting results were compared with the results obtained using the empirical formula for pressure drop.

The pressure drop of the pipe flow is mainly composed of two parts: major and minor head loss. The pipe flow pressure drop of a U-tube CMF is the sum of these two parts multiplied by the acceleration of gravity and fluid density:

(24) $$\Delta p=\rho_{f}g\left(h_{major}+h_{minor}\right)$$

where $\rho_{f}$ is the fluid density, g is the acceleration of gravity, $h_{major}$ is the major head loss, and $h_{minor}$ is the minor head loss. Major head loss is obtained using the Darcy–Weisbach equation as follows:

(25) $$h_{major}=f_{d}\frac{L}{D}\frac{U^{2}}{2g}$$

where $f_{d}$ is the Darcy friction factor. To facilitate the curve fitting, the approximate Colebrook equation, which was developed by Moody [17], was used; thus, the following equation is proposed by assuming that the surface roughness of the pipe wall (ϵ) is 0:

(26) $$f_{d}=0.0055\left[1+{\left(20000\epsilon +\frac{10^{6}}{Re}\right)}^{1/3}\right].$$

**Figure 20:**
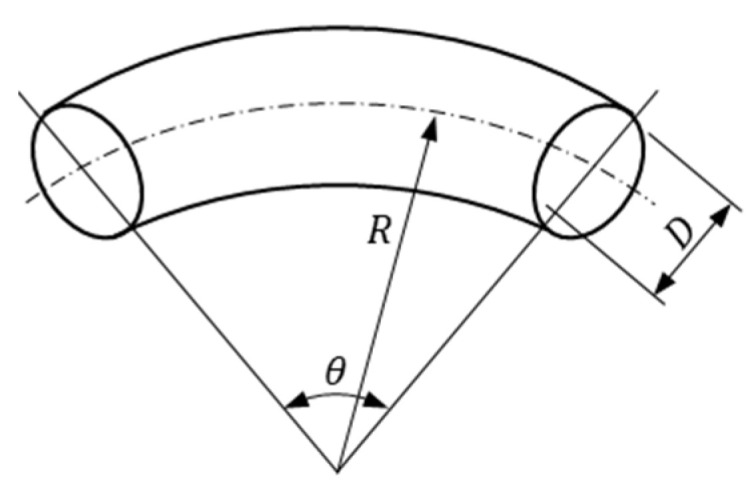
Schematic of the bend.

Minor head loss is caused by the geometric structure of the pipe flow system. This study mainly discusses the pressure drop caused by the minor head loss of the pipe elbow. The schematic of the bend is illustrated in Figure 20. The following formula, which is primarily based on the formula provided by Idelchik [18], can be used to calculate the pipe flow pressure drop due to the bent pipe:

(27) $$\Delta p=0.0175\frac{5}{Re^{0.45}}{\left(\frac{D}{2R}\right)}^{0.275}\theta \frac{R}{D}\frac{\rho_{f}U^{2}}{2}.$$

**Figure 21:**
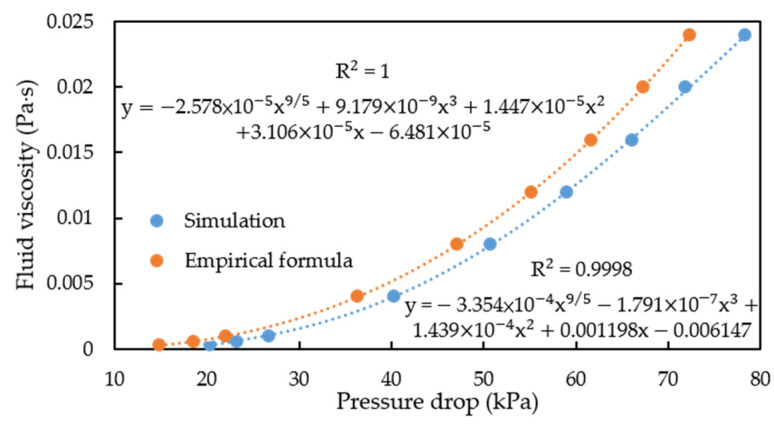
Relationship between fluid viscosity and the pipe flow pressure drop.

As shown in Figure 21, the results obtained through simulation and with the empirical formula exhibit identical trends. The pressure drop–fluid viscosity relationship was found through polynomial function fitting, which is an empirical method for determining the power term. The fitting result for the COMSOL simulation was satisfactory, and the coefficient of determination (*R*^2^) was found to be nearly 1. Therefore, in this research, COMSOL finite-element simulation and polynomial fitting could be used to establish the relationship between fluid viscosity and the pipe flow pressure drop.

### 5.2. Fluid Viscosity Estimation Through Driving Current

**Figure 22:**
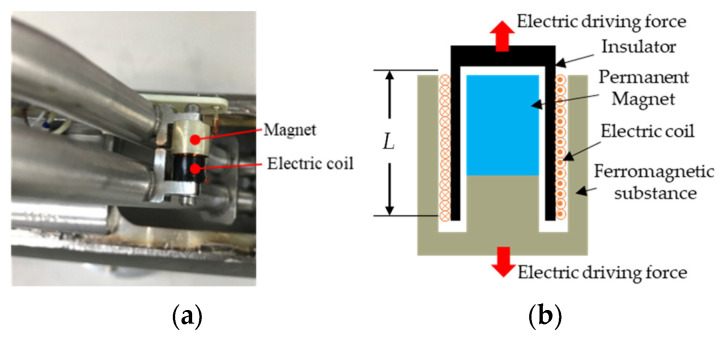
The voice coil actuator/sensor: (**a**) the photo; (**b**) the schematic.

A dual U-tube CMF contains two identical U-tubes connected in parallel. A voice coil actuator (Figure 22) mounted on the U-tube midpoint drives the U-tubes to oscillate symmetrically normal to the U-tube plane with identical frequencies and constant amplitudes. According to Paidoussis and Issid [19], the influence of the fluid shear force in the pipe flow on the lateral displacement (W) of the pipe can be deduced using the equation of motion obtained by ignoring the axial force:

(28) $$\left(m_{t}+m_{f}\right)\frac{\partial^{2}W}{\partial t^{2}}+m_{f}{\left(\frac{\partial }{\partial t}+U\frac{\partial }{\partial x}\right)}^{2}+c_{f}\frac{\partial W}{\partial t}+EI\frac{\partial^{4}W}{\partial x^{4}}=0$$

where $c_{f}$ is the viscous damping coefficient due to the shear force, E is the Young’s modulus of the tube, I is the inertia moment of the tube’s cross-section area, $m_{t}$ is the mass of tube per unit length, $m_{f}$ is the mass per unit length of the fluid inside the tube, U is the flow velocity of the fluid relative to the oscillatory tube, and W is the transverse deflection of the tube. The higher the fluid viscosity, the higher is the shear resistance of the fluid and the higher is the damping effect when the flow-tube structure vibrates. Therefore, to maintain flow-tube amplitude constant, the driver’s drive current must be adjusted according to the fluid viscosity automatically, in other words, the driving current of a CMF is not constant. Theoretically, the fluid viscosity can be deduced from the driving current of the voice coil actuator.

We divided the relationship between driving current and fluid viscosity into two parts. The current–driving force relationship was established on the basis of the current magnetic effect, whereas the driving force–viscosity relationship was established through curve fitting with the simulation results. The driving force of a voice coil actuator was usually proportional to the driving current [20], namely:

(29) $$F_{0}=kBLIN$$

where $F_{0}$ is the driving force, *k* is force constant, *B* is magnetic flux density, *L* is the length of the coil, *I* is current, N is the number of turns.

**Figure 23:**
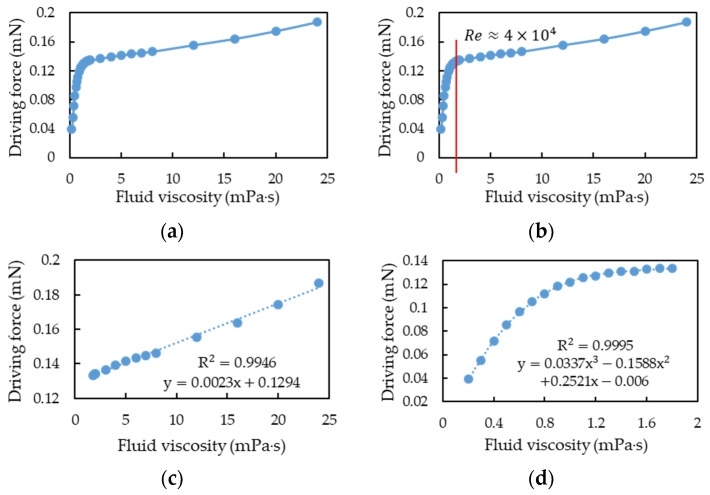
Simulation of fluid viscosity vs. driving force when the flow rate was 1 kg/s: (**a**) the simulation result. (**b**) the cutoff point of linear and nonlinear region was at *Re* = 40,000. (**c**) the linear function by curve fitting as *Re* < 40,000. (**d**) the cubic polynomial function by curve fitting as *Re* > 40,000.

The simulation method was used to estimate the driving force required to maintain the motion sensor positions at a vibration amplitude of 0.5 μm under different fluid viscosities. Finally, we found the relationship between driving force and fluid viscosity through curve fitting of the experimental results with the simulation results. The mass flow rate was set as the mid-value (1 kg/s) of the CMF design specification for simulation, and the viscosity range was set as 0.2–24 mPa∙s. The simulation results are illustrated in Figure 23a. In this study, a cubic polynomial function and a linear function were used to fit the two nonlinear and linear intervals, respectively, and the coefficient of determination (*R*^2^) was used to judge the quality of the fit and the cutoff point of the two ranges. The results indicate that with a dimensionless *Re* of 40,000 as the cutoff point (Figure 23b) the fitting results for the linear interval (Figure 23c), and the nonlinear range (Figure 23d) were satisfactory (*R*^2^ ≈ 1).

**Figure 24:**
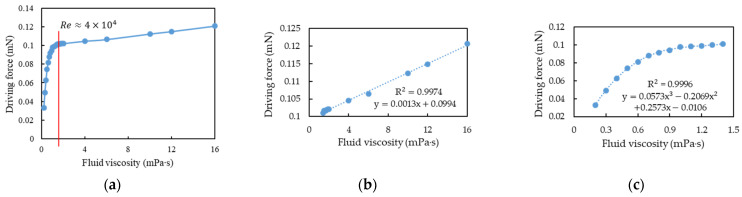
Simulation of fluid viscosity vs. driving force when the mass flow rate was 0.8 kg/s: (**a**) the cutoff point of linear and nonlinear region was at *Re* = 40,000; (**b**) the linear function by curve fitting as *Re* < 40,000; (**c**) the cubic polynomial function by curve fitting as *Re* > 40,000.

**Figure 25:**
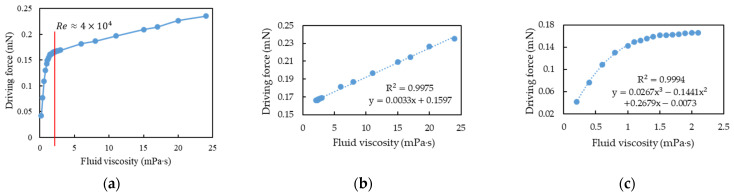
Simulation of fluid viscosity vs. driving force when the mass flow rate was 1.2 kg/s: (**a**) the cutoff point of linear and nonlinear region was at *Re* = 40,000; (**b**) the linear function by curve fitting as *Re* < 40,000; (**c**) the cubic polynomial function by curve fitting as *Re* > 40,000.

To verify whether the boundary between the linear and nonlinear intervals changed or was at *Re* = 40,000 under different mass flow rates, two sets of mass flow rates (0.8 and 1.2 kg/s) were used for simulation analysis. After fitting analysis, the simulation results of fluid viscosity and driving force at 0.8 kg/s were also segmented at *Re* = 40,000 (Figure 24a). The linear and cubic polynomial fitting results at the aforementioned mass flow rate are illustrated in Figure 24b,c, respectively. When the mass flow rate was 1.2 kg/s, the simulation results of fluid viscosity and driving force were also segmented at *Re* = 40,000, (Figure 25a). The linear and cubic polynomial fitting results at the aforementioned mass flow rate are presented in Figure 25b,c, respectively. From the simulation results of the three flow rates, namely 0.8, 1, and 1.2 kg/s, and the results of segmented curve fitting, we found that the different flow rates were based on the same *Re* segment as the linear and cubic fitting curves were.

**Figure 26:**
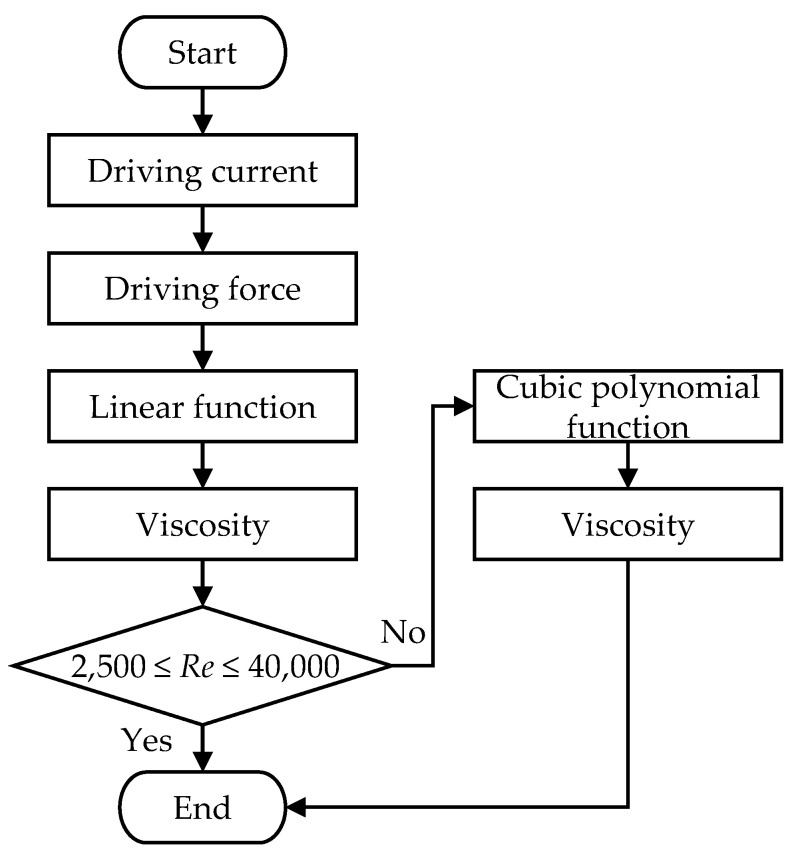
The flowchart of deducing the fluid viscosity from the driving force.

In summary, for each flow rate, one can obtain two empirical formula of driving force and fluid viscosity, one is a linear function and another is a cubic polynomial function; furthermore, the linear function is applicable for *Re* < 40,000 while the cubic polynomial function is for *Re* > 40,000. If the driving force is known, then the fluid viscosity may be deduced through the empirical formula. However, in practice, the driving current is known but not the driving force. Therefore, a transfer function, like Equation (29), between the driving current and the driving force is required. Figure 26 shows the flowchart of how to deduce the fluid viscosity from the driving current. Firstly, the driving force is obtained from the transfer function of the driving force and driving current of the voice coil actuator. Secondly, assume the linear function is applicable, it is easy to calculate the fluid viscosity by substituting the driving force into the linear empirical formulas. Then, use the result to check whether *Re* is less than 40,000; if it is, the process ends, otherwise, the driving force is substituted into the cubic polynomial empirical formula to solve for the fluid viscosity, and the process ends.

### 5.3. Motion Sensor Positions

**Figure 27:**
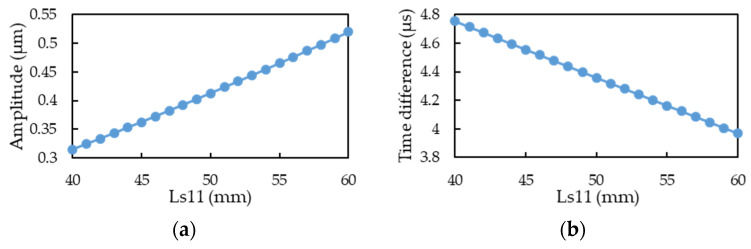
The influence of motion sensor position on: (**a**) the vibration amplitude at the position of motion sensor; (**b**) the phase difference to the output signals of the motion sensors.

The two CMF motion sensors are symmetrically located on both sides of the CMF flow tube. In signal processing, the magnitude and phase difference of the output signals influence measurement accuracy. In this study, we used COMSOL to simulate the influence of the motion sensors’ installation position on the magnitude and phase difference of the output signals. The simulation results are presented in Figure 27, where the horizontal axes represent the motion sensor installation positions (Ls11; see Figure 2 for motion sensor installation positions). The Ls11 position of the original commercial CMF is located at approximately 50.7 mm; therefore, we used this position as the initial installation position, with a deviation of ±10 mm. From the simulation results, we found that when the installation position of the motion sensor was closer to the middle of the flow tube, the phase difference between the two signals became greater and the signal amplitude was smaller. When the sensor position is closer to the middle of the flow tube, the signal amplitude increases linearly. Therefore, when choosing the motion sensor installation position, please consider the acceptable signal amplitude and phase difference range for signal processing.

### 5.4. Flow Splitter Design

**Figure 28:**
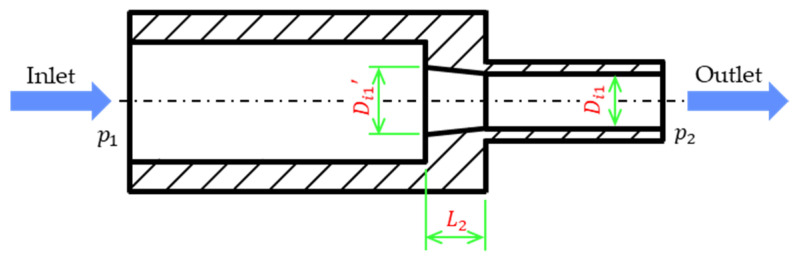
Cross-sectional schematic of the conical splitter structure.

In the dual U-tube CMF, a single pipe is split into two sections by using a splitter. The structure of this splitter causes severe pressure loss. According to fluid pressure distribution illustrated in Figure 28, the splitter structure causes a 50% pressure drop in the entire CMF. In general, when the total pressure drop in a CMF is ≤150 kPa, the pressure loss in the pipeline is excessively large and the pump exerts an increased amount of energy to increase fluid pressure. Consequently, the cost of fluid transportation increases [21]. Therefore, in this study, through COMSOL simulation, we designed a splitter structure to reduce pressure loss in the pipeline and alleviate the pressure drop.

**Figure 29:**
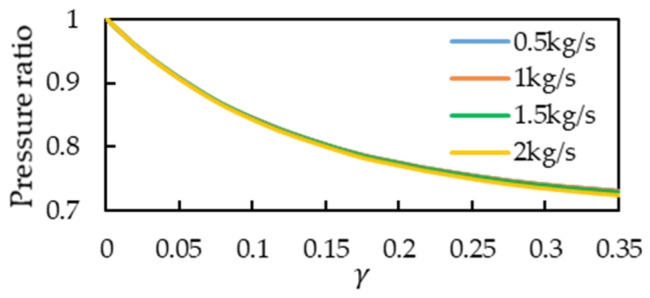
Simulation results of the conical splitter structure.

**Figure 30:**
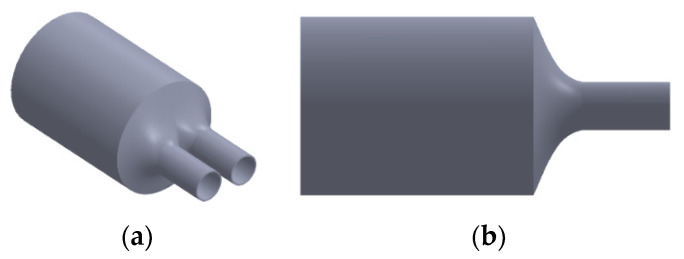
Taper curved conical splitter structure: (**a**) isometric and (**b**) front views.

The design of the first split structure is conical. The schematic cross-sectional view of this structure is presented in Figure 28. The taper of the conical structure is defined as follows:

(30) $$\gamma =\left({D_{i1}}^{\prime }-D_{i1}\right)/L_{2}$$

when the original length of the split structure is $L_{2}$ and the original distance between the two tubes and the diameter of the flow tube $D_{i1}$ are not changed, only the base diameter of the cone ${D_{i1}}^{\prime }$ is adjusted for simulation analysis and the pressure drop is $p_{1}-p_{2}$. The higher the flow, the higher is the pressure drop. Therefore, observing the pressure drop of a low flow was difficult. Consequently, as shown in Figure 29, the pressure ratio, which is dimensionless and defined as the pressure drop after the structural design divided by the pressure drop without structural design, is represented on the vertical axis. Moreover, in the simulation, the adjustable maximum taper was 0.35, and the minimum pressure ratio at each flow rate was approximately 0.73. The simulation included a second splitter that had a tapered structure with a curved surface (Figure 30), and the pressure ratio for this structure was approximately 0.72.

## 6. Conclusions

This paper simulates the fluid-structure coupling dynamics of a dual U-tube Coriolis mass flowmeter by the use of COMSOL’s finite-element simulation package and validates the simulation through experiments in a TAF certified flow testing factory. Some important design considerations are addressed as the following:

- The fluid density is inversely proportional to the square of resonant frequency. The deviation of the simulation from the experiment is only 3% and regular. The slight deviation may come from the frequency-dependent dynamic water stiffness and damping and the mass density of the solid-air-water mixture.
- The accurate calculation of the boundary layer is essential to analyze the fluid–structure coupling simulation of the CMF accurately, but accurate calculation of the boundary layer requires very fine mesh and very large numerical computation loading.
- In a factory, production lines typically include many complicated pipelines. Therefore, the installation angle of CMF must be adjusted according to the actual allowable space. A guideline is that the flow tube cannot be placed upwards, because air is lighter then water, otherwise the air will accumulate in the flow tube and cause measurement error. CMF can install any deflection angle under the premise that the flow tubes are oriented downward.
- The relationship between the mass flow rate and the time difference of the two motion sensors’ output is a linear function. The structural imbalance of the flow tubes only introduces an offset of such relationship, namely change the constant term of the linear function, under the premise that the unbalance mass is not large enough to change the resonant frequency of the CMF significantly.
- The fluid viscosity can be deduced from the pressure drop of between the inlet and outlet of CMF, because the higher the fluid viscosity, the higher is the shear of the pipe wall, which causes changes in the pipe flow pressure drop. For CMF, the pressure drop is the sum of major and minor head loss multiplied by the gravitational acceleration and fluid density.
- Two empirical formula of driving force and fluid viscosity are proposed, one is a linear function and another is a cubic polynomial function; furthermore, the linear function is applicable for *Re* < 40,000 while the cubic polynomial function is for *Re* > 40,000. If the driving force is known, then the fluid viscosity may be deduced through the empirical formula. However, in practice, the driving current is known but not the driving force. Fortunately, the driving force of a voice coil actuator is proportional to the driving current. If the transfer function between the driving current and the driving force is known, then the fluid viscosity can be deduced from the driving current of the voice coil actuator through the two empirical formula of driving force and fluid viscosity. Firstly, the driving force is obtained from the transfer function of the driving force and driving current of the voice coil actuator. Secondly, assume the linear function is applicable, it is easy to calculate the fluid viscosity by substituting the driving force into the linear empirical formulas. Then, use the result to check whether *Re* is less than 40,000; if it is, the process ends, otherwise, the driving force is substituted into the cubic polynomial empirical formula to solve for the fluid viscosity, and the process ends.
- When the motion sensor is closer to the middle of the flow tube, its output signal increases linearly, while the time difference of signals outputted by the two motion sensors decreases linearly. Therefore, there is trade-off on the position of the motion sensors for the magnitude and time difference of the signal.
- If the pressure loss of pipe flow is too large, it is an effective method to design the flow splitter’s conical structure.
- The authors have developed a dual U-tube design application (App) based on COMSOL application development platform. Users can quickly evaluate their design through input the geometric and material parameters of the structure, the type of fluid, and the measurement specifications. The present application can significantly shorten product design and manufacturing time. The user interface is shown in Appendix A.

## Figures and Tables

**Table 1:** CMF dimensions.

Symbol	Values	Symbol	Values
Df	124.00 mm	Df1	19.50 mm
Di	26.50 mm	Di1	9.00 mm
Do	37.00 mm	Do1	10.00 mm
H	31.50 mm	LB	13.60 mm
Lf	13.50 mm	L1 = L3	48.50 mm
L2 = L4	10.20 mm	L11 = L13	83.00 mm
L12 = L14	28.70 mm	Ls11 = Ls12	50.70 mm
Rf	45.00 mm	R11	31.65 mm
R12 = R13	31.70 mm	θ11 = θ14	76°
θ12 = θ13	76°	θ3 = θ4	53°
θ5 = θ6	22°	W	17.00 mm

**Table 2:** Materials and masses of motion sensors and driver.

SAE 316L Stainless Steel	
Young’s Modulus (GPa)	193
Poisson Ratio	0.275
Density (kg/$m^{3}$)	8000
Melting Point (°C)	1400
Thermal Expansion (1/K)	$15.9\times 10^{-6}$
Thermal Conductivity (W/m⋅K)	16.3
Electrical Resistivity (Ω⋅m)	$0.074\times 10^{-6}$
Driver mass (g)	8.00
Motion sensor mass (g)	5.40
**Water at 25 °C**	
Density (kg/$m^{3}$)	997.05
Viscosity (mPa⋅s)	0.89

**Table 3:** The constants of k-ε turbulence model.

Constants	Values
*C_μ_*	0.09
*C*_1*ε*_	1.44
*C*_2*ε*_	1.92
*σ_k_*	1
*σ_ε_*	1.3

**Table 4:** The constants of SST turbulence model.

Constants	Values
*α*_1_	0.56
*α*_2_	0.44
$\beta_{0}^{*}$	0.09
*β*_1_	0.075
*β*_2_	0.0828
*σ_k_*_1_	0.5
*σ_k_*_2_	1
*σ_ω_*_1_	0.5
*σ_ω_*_2_	0.856

**Table 5:** Fluid density experiment and simulation results.

Experiment						Simulation	
wt %	Density by CMF (kg/m^3^)	Density by Meter (kg/m^3^)	Absolute Error (kg/m^3^)	Frequency (Hz)	Phase (mrad)	Density (kg/m^3^)	Frequency (Hz)
0.00	998.0	997.1	0.9	327.56	0.006	995	317.39
1.00	1005.0	1004.3	0.7	327.25	0.002	1005	316.96
2.00	1012.1	1011.2	0.9	326.94	0.000	1015	316.53
4.00	1024.6	1025.4	0.8	326.38	0.006	1025	316.10
6.00	1039.3	1039.6	0.3	325.74	0.008	1035	315.67
8.00	1051.8	1053.8	2.0	325.20	0.001	1045	315.24
10.00	1068.8	1068.5	0.3	324.46	0.001	1055	314.82
12.00	1083.3	1083.5	0.2	323.84	0.009	1065	314.40
14.00	1099.4	1098.9	0.5	323.15	0.001	1075	313.98
16.00	1114.7	1115.1	0.4	322.5	0.001	1085	313.56
18.00	1128.7	1130.5	1.8	321.91	0.001	1095	313.14
20.00	1146.9	1147.2	0.3	321.15	0.004	1105	312.73
22.00	1161.8	1162.3	0.5	320.53	0.005	1115	312.31
24.00	1180.4	1181.9	1.5	319.76	−0.002	1125	311.90
26.00	1192.6	1192.9	0.3	319.26	0.003	1135	311.49
						1145	311.08
						1155	310.67
						1165	310.27
						1175	309.86
						1185	309.46
						1195	309.06

**Table 6:** Experimental and simulation results for mass flow rate.

Experiment			Simulation	
Flow Rate (kg/s)	Time Difference (μs)	Flow Rate (kg/s)	Time Difference (μs)	
			*k-ε* Method	SST Method
0.00	0.00	0.0	0.00	0.00
0.14	0.78	0.2	0.67	0.99
0.28	1.60	0.4	1.36	1.86
0.56	3.22	0.6	2.03	2.69
0.84	4.84	1.0	3.43	4.34
1.12	6.46	1.5	5.21	6.44
1.40	8.08	2.0	6.93	9.02
1.69	9.71			
1.94	11.18			

**Table 7:** The output voltage amplitudes of imbalance experiment.

	Output Voltage of Sensor 1 (S1)	Output Voltage of Sensor 2 (S2)	S1-S2
No additional mass	72.114 mV	74.264 mV	−2.150 mV
Additional mass below Sensor 1	75.614 mV	74.728 mV	0.886 mV
Additional mass below Sensor 2	69.724 mV	74.732 mV	−5.008 mV

**Figure A1:**
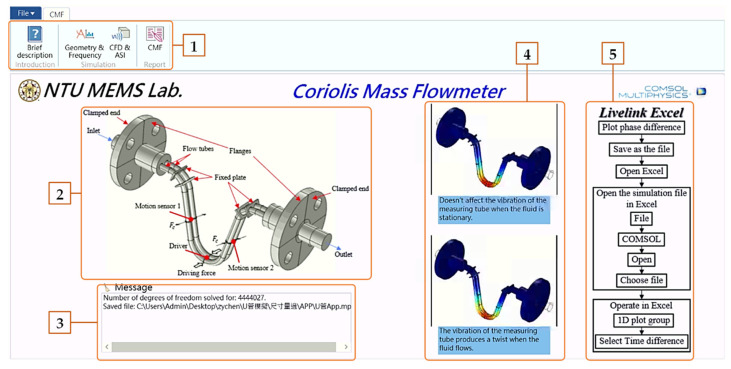
The simple introduction page of the App.

**Figure A2:**
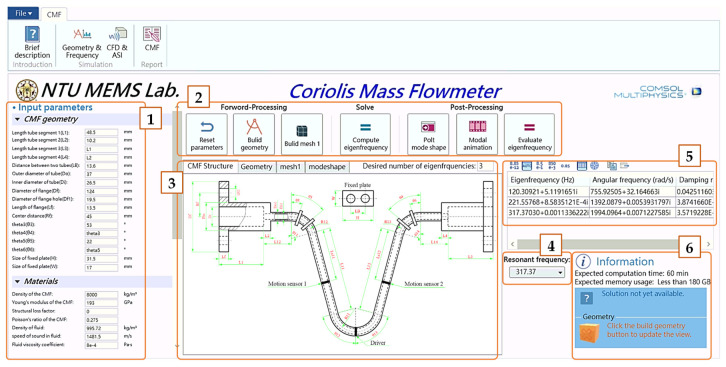
The geometry establishment and resonance frequency simulation page of the App.

**Figure A3:**
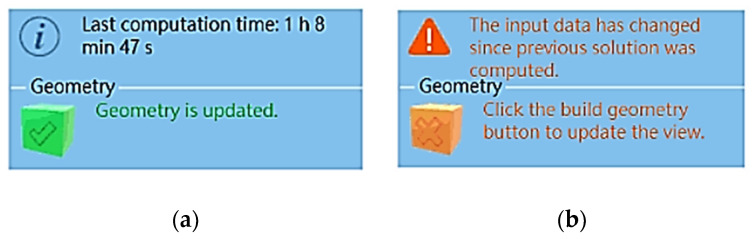
Message window: (**a**) for geometry creation and successful solution; (**b**) for reminding the parameters have been changed.

**Figure A4:**
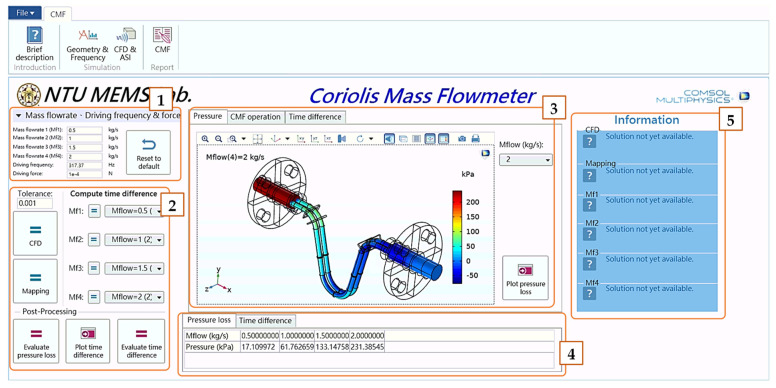
The fluid dynamics and simulation of the fluid-structure interaction page of the App.

**Figure A5:**
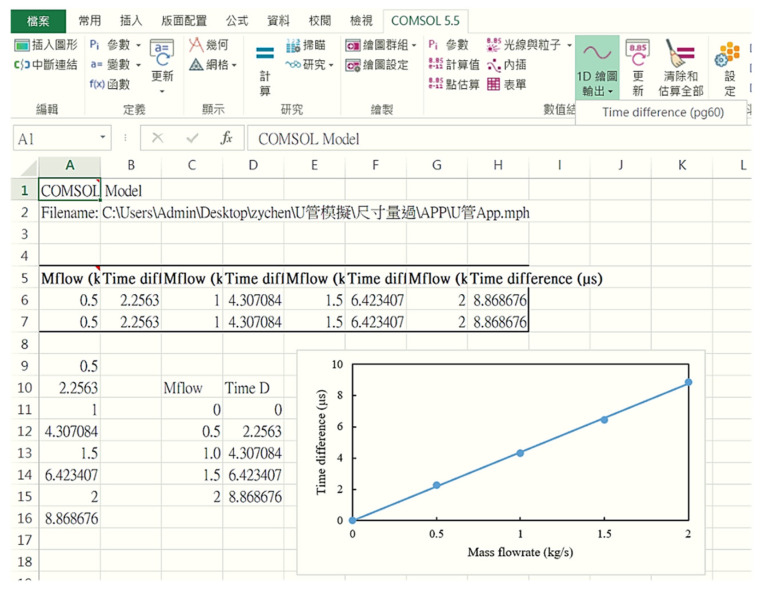
Simulation results imported from the COMSOL app into an MS Excel spreadsheet.

## Data Availability

Not applicable.

## Appendix A. The User Interface of the COMSOL Simulation App

In this section, we introduce the function and operation of a self-developed COMSOL simulation app. This app is mainly divided into three pages: (1) simple introduction, (2) geometric establishment and resonance frequency simulation, and (3) computational fluid dynamics and simulation of the fluid–structure interaction. The functions of the three pages are explained in the following subsections.

### Appendix A.1. Simple Introduction Page

The “Simple Introduction” page (Figure A1) has the following functions:

(1) Pagination switching buttons: Positioned from left to right are the front cover of the simple introduction, geometric establishment and resonance frequency simulation, and computational fluid dynamics and simulation of the fluid–structure interaction.
(2) Introduction to the dual U-tube CMF: Roughly describes the structure and boundary conditions of the simulated CMF with images.
(3) Message window: Display the operation record of the simulation app.
(4) Operation of the dual U-tube CMF: Aids the user in roughly understanding CMF operation when fluid is flowing or not flowing. The part above the text description is presented in the animation.
(5) Steps to connect to MS Excel: A flowchart providing users with an idea of how to connect COMSOL to MS Excel and process data.

### Appendix A.2. Geometry Establishment and Resonance Frequency Simulation Page

The “Geometry Establishment and Resonance Frequency Simulation” page (Figure A2) has the following functions:

(1) Input parameters: Includes the geometric and material parameters of the CMF.
(2) Button area: Roughly divided into preprocessing, solving, and postprocessing. Placed from left to right are resetting parameters to set values, create geometry, create meshes, calculate characteristic frequencies, draw modals, and play modal animations.
(3) Visualization results: Displays geometry and mesh creation results as well as simulation results. In the upper right corner, the user is allowed to input freely and solve several characteristic frequencies.
(4) Dropdown list of frequency: The simulation result of the CMF resonance frequency can be selected to plot the mode of the frequency.
(5) Display calculated value: Displays all the characteristic frequency values.
(6) Message window: Displays the status of geometry creation and the solution. Separate message windows are displayed when the geometry structure of the dual U-tube CMF is created and when the solution is completed. The information on the geometry creation and solution success is displayed to the user, Figure A3a. After the solution’s geometry is completed, any further changes to the parameters are displayed in separate message windows. Another message window reminds the user that parameters have been changed, Figure A3b.

### Appendix A.3. Computational Fluid Dynamics and Simulation of the Fluid–Structure Interaction Page

The “Fluid Dynamics and Simulation of the Fluid-Structure Interaction” page (Figure A4) has the following functions:

(1) Input parameters: Displays parameters for mass flow rate, driving frequency, and driving force as well as a parameter reset button.
(2) Solve and postprocessing button: The solve button enables the study of computational fluid dynamics, the marginal smoothening of the solution, and the simulation of the fluid–structure interaction to solve the phase difference. The above three steps must be solved in order. Also, users can input the iterative tolerance of CFD. Post-processing includes calculating the pipe flow pressure loss, plotting the relationship between mass flow rate and the phase difference, and calculating the phase difference.
(3) Display of simulation results: Includes CMF pressure loss and operation as well as the relationship between mass flow rate and phase difference.
(4) Display of calculated value: Displays the pressure loss and phase difference under different mass flow rates.
(5) Message window: Displays the status of the solution. Its function is the same as the message window on the previous page.

Finally, as depicted in Figure A5, the simulated file can be opened in a preset Excel spreadsheet, and the phase difference–mass flow rate relationship is automatically plotted.
